# Supplementary material for: Migration rather than proliferation transcriptomic signatures are strongly associated with breast cancer patient survival
Source: Sci Rep. 2019 Jul 29;9:10989. doi: 10.1038/s41598-019-47440-w (PMC6662662; doi:10.1038/s41598-019-47440-w)
Supplement: Supplementary file 1 — Supplementary note and figures [file 41598_2019_47440_MOESM1_ESM.docx]

**Supplementary note and figures for “Migration rather than proliferation transcriptomic signatures are strongly associated with breast cancer patient survival”**

Nishanth Ulhas Nair^1,2,#^, Avinash Das^3,4,#^, Vasiliki-Maria Rogkoti^5^, Michiel Fokkelman^5^, Richard Marcotte^6,7^, Chiaro G. de Jong^5^, Esmee Koedoot^5^, Joo Sang Lee^1,2^, Isaac Meilijson^8^, Sridhar Hannenhalli^1^, Benjamin G. Neel^6,9,10^, Bob van de Water^5^, Sylvia E. Le Dévédec^5^, Eytan Ruppin^1,2,11,12,*^

1 – Center for Bioinformatics and Computational Biology, University of Maryland, College Park, Maryland 20742, USA.
2 – Cancer Data Science Lab, National Cancer Institute (NCI), National Institutes of Health (NIH), Bethesda, USA.

3 – Department of Biostatistics and Computational Biology, Harvard School of Public Health, Boston, USA.

4 – Massachusetts General Hospital Cancer Center, Harvard Medical School, Boston, USA.
5 – Division of Drug Discovery and Safety, LACDR, Leiden University, Leiden, the Netherlands.

6 – Princess Margaret Cancer Centre, University Health Network, Toronto, ON M5G 1L7, Canada.

7 – National Research Council Canada, Montreal, Canada.

8 – Department of Statistics and Operations Research, School of Mathematical Sciences, Tel Aviv University, Tel Aviv 69978, Israel.

9 – Laura and Isaac Perlmutter Cancer Centre, NYU-Langone Medical Center, NY 10016, USA.

10 – Alexandria Center for Life Science, New York, NY 10016, USA.

11 – The Blavatnik School of Computer Science, Tel Aviv University, Tel Aviv 69978, Israel.

12 – Lead Contact

# – contributed equally.

* – corresponding author email: eytan.ruppin@nih.gov

Current affiliations – NUN, JSL is (2); ER is (2,11); RM is (7); BGN is (9,10).

**Supplementary Note**

**CellToClinic Predictors**

CellToPhenotype predictors consists of two expressions based supervised regression – one for predicting cell migration and other for predicting cell proliferation. Each predictor was trained on *in vitro* cell migration or proliferation as the dependent variable and gene expression of cell lines as the independent variables in the regression. The gene expression data was obtained from the Cancer Cell Line Encyclopedia project^1^. Gene expression data was transformed to a standard normal distribution across genes and samples.

CellToPhenotype adopts two level feature selections to reduce testing error. First, 2448 genes that are significantly associated (FDR<0.01 using Cox regression) with patient survival (in an independent dataset – METABRIC). The “survival” package in R was used^2^ to determine the association. Second, CellToPhenotype uses LASSO (least absolute shrinkage and selection operator) regressor to regularize the predictor that enables a data-driven feature selection using a cross-validation. A five-fold cross validation procedure to compute the minimum λ value for LASSO. The “glmnet” package in R was used to perform regression^3^. To increase the generalizability of predictors, the LASSO regression selects only subset of genes as the gene signatures of the CellToPhenotype predictors (L1-norm regularization). That is, LASSO selects only a small number of the 2448 genes (during each iteration of LASSO) for constructing the final predictor. Note, both feature selection steps were conducted in dataset independent of the testing set on which performance of CellToPhenotye was evaluated. This ensures an unbiased evaluation of predictive power of CellToPhenotype.

Applying the predictor learned above to the gene expression of breast cancer tumor samples, we can predict migratory and proliferation level for each sample/individual. To obtain a robust estimate of levels, we iterate this procedure 50 times and take the median value of the migration and proliferation levels as the final estimates. (For each iteration, LASSO chooses a few genes for model training. The frequency value mentioned next to each gene in Tables S3(a-d) is the number of times a gene has been selected for model training.)

We explain LASSO regression method in more detail below:

Linear regression is a linear approach to model a relationship between a dependent variable and one or more independent variables^4^. LASSO is a shrinkage and selection method for linear regression^5^. LASSO was introduced to avoid overfitting by selecting only a subset of the provided covariates in the final model rather than using all of them. It performs both variable selection and regularization in order to improve prediction accuracy. Regularization is a form of regression which shrinks the coefficients of the independent variables to zero and avoids overfitting. LASSO uses an L1-regularization technique, which adds an absolute value of coefficient as penalty term to the loss function. The object of LASSO to minimize the following function:


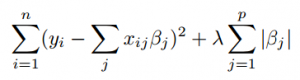


where y is the dependent variable, x is the independent variable, β is the coefficient of regression (with constraint Σβ_j_ ≤ *s*), and λ is the tuning parameter. Some of the βs are shrunk to zero due to the regression.

One disadvantage of L1-regularization is that it cannot help with multicollinearity. However, that is not a concern in our case the data we work with does not suffer from a multi-collinearity problem.

**Multi-collinearity check**

We use a LASSO-based regression (along with cross validation) and LASSO can handle co-variance among features^6,7^. In fact, it has been experimentally shown that cross validation plus LASSO (with much of supporting theory) works well with co-varying features. However, LASSO suffers from a multi-collinearity problem. Here we show that our data do not suffer from multicollinearity. When input features are multi-collinear, in essence, LASSO can arbitrarily select one of the collinear features. To test for multi-collinearity in the model training data, we computed the Variance Inflation Factor (VIF) in the training data. If VIF values are greater than 10, then the data may have multi-collinearity issues^8^. We got VIF values ranging from 1.2 to 4.06 (median = 1.43), thereby showing that our data does not suffer from multicollinearity.

In the case of covariance between variables in linear regression, the estimated coefficient from the linear model would have high variance but final predicted values of phenotype are not affected by this. To explicitly show this, we now conduct robustness analysis of the LASSO-based predictor. The LASSO based predictors give consistent results over multiple iterations, showing that the LASSO based predictions give quite consistent results irrespective of any covariance among features. Figure S5 shows the consistency of the predicted migration and proliferation values for one patient in the TCGA data (run for 10 iterations).

**Estimating effect of migration and proliferation on survival**

The effect of migration and proliferation levels (models built using 40 common breast cancer cell lines) on patient survival was estimated 1043 breast cancer patients TCGA data as follows. To check the association of the predicted migration with patients’ survival we fit following Cox regression:

$$\begin{aligned} Survival \sim migration+strata\left( \mathrm{race} \right)+age+GII \end{aligned}$$

Patient survival is known to be confounded by age, race, and genomic instability. Including these factors in our Cox regression model, we systematically control for their confounding effect on survival. Genomic instability index (GII) measures the relative amplification or deletion of genes in a tumor based on the somatic copy number alteration (SCNA). Let $p_{i}$ be the absolute of log ratio of SCNA of gene *i* in a sample relative to normal control, GII of the sample is^9^:

$$GII=1/N\sum_{1}^{N} {I(p}_{i}>1)$$

where “I” is the indicator function.

Strata (race) in the above model implies Cox regression was conducted in each patient stratification based on race separately and likelihood were combined. We repeated the procedure for 10 iterations, and median coefficients (risk factor) of migration were computed. The association of survival with proliferation was estimated similarly.

To estimate the relative contribution of migration and proliferation to predict patient survival we fit following Cox regression, which also controls for age, race, and genomic instability:

$$\begin{aligned} Survival \sim migration+proliferation+strata\left( \mathrm{race} \right)+age+GII \end{aligned}$$

We used the “lrt” function in R to do likelihood ratio test between two Cox regression models.

Each Kaplan Myer (KM) analysis was done by comparing the migration/proliferation levels of on the top 25 percentile of patients with bottom 25 percentile patients (Figure 3b).

**Control experiments**

Since we used survival genes as features, we wanted to check if the results we obtain (Figures 2, 3) is purely because of this, and if the LASSO regression (in the CellToPhenotype predictors) holds any value. So as a control experiment, we trained migration and proliferation based models using LASSO regression as before (using 2448 genes associated with survival). Then we randomly shuffled these survival-significant genes and their regression coefficients while predicting migration and proliferation levels. This is basically the same as taking a random linear combination of the gene expression of the survival-significant genes to predict migration and proliferation levels. This was done for 20 iterations. We predict migration and proliferation levels for each iteration. The results of the control experiments for various phenotypes are given below.

Paired Wilcoxon rank-sum test between 110 tumors and matched normal samples for both the predicted migration and proliferation did not show any significant difference (P<0.6 and P<0.47 respectively). Mean p-value between the two groups over various iterations is shown in brackets.

We did not find any significant increase in predicted migration levels from stage I and stage II (Wilcoxon rank sum test, P<0.45); and from stage II to stage III-IV (P<0.45). Predicted proliferation levels also did not increase from stage I to stage II (P<0.41) and stage I to stage III-IV (P<0.39).

We see that predicted migration levels did not increase from grade 1 to grade 2 (Wilcoxon rank-sum, P<0.41), and from grade 2 to grade 3 (P<0.33), and proliferation levels did not increase from grade 1 to grade 2 (P<0.36), and from grade 2 to grade 3 (P<0.36).

We do not find any significant association of predicted migration levels with patient survival (risk factor = 0.067, P<0.24) or between proliferation and survival (risk factor = -0.0021, P<0.24). Mean value of the risk factor over various iterations is shown.

These results show that the results that we obtain (Figures 2, 3) are not only due to the feature selection, as the random linear combination of the expression of these selected genes to predict migration and proliferation levels do not yield good results.

**Survival analysis**

When we repeated the survival analysis in TCGA data by randomly sampling 30 cell lines (iterated 10 times, each time there is a random sampling), we did not see any survival prediction capability for both migration and proliferation.

**Correlations**

Spearman correlations between KD-migration-score and predicted migration levels (CellToPhenotype) and experimentally measured migration values are given in Figure S1. Similar analyses were conducted for proliferation (Figure S1). KD-migration-score has a high correlation with the predicted migration levels (Spearman ρ = 0.83, P<4.36e-11, Figure S1a) and experimentally measured values (Spearman ρ = 0.79, P<1.9e-9, Figure S1b). KD-proliferation-scores are highly correlated with both the predicted proliferation levels (Spearman ρ = 0.75, P<3.07e-8, Figure S1c) and the experimentally measured proliferation values (Spearman ρ = 0.82, P<2.58e-10, Figure S1d). We also checked cross correlation values between KD-migration-score and experimentally-measured proliferation values. Spearman correlation between KD-migration-score and experimentally-measured proliferation values (Spearman ρ = 0.47, P<0.0023) is comparatively low. Similarly, Spearman correlation between KD-proliferation-score and experimentally measured migration values (Spearman ρ = 0.55, P<0.00024) is also comparatively low.

**Subtype analysis**

TNBC patients exhibit higher predicted migration than Luminal A patients (ANOVA, P<0.0024), Luminal B patients (ANOVA, P<0.0059), and Her2 positive patients (ANOVA, P<0.049). The mean value of the predicted migration also showed significant differences between all 4 subtypes (ANOVA using 4 groups, P<0.015).

TNBC patients exhibit higher predicted proliferation than Luminal A patients (ANOVA, P<2.2e-16), Luminal B patients (ANOVA, P<2.2e-16), and Her2 positive patients (ANOVA, P<0.0014). The mean value of the predicted proliferation also showed very significant differences between all 4 subtypes (ANOVA using 4 groups, P<2.2e-16).

Luminal A has significantly lower predicted proliferation compared to Luminal B (ANOVA, P<0.0015).

We, however, did not find a statistically significant difference between predicted migration levels of Luminal A patients and Luminal B patients (ANOVA, P<0.88), and between predicted migration levels of Luminal A patients and Her2 positive patients (ANOVA, P<0.58).

**Robustness of cell migration measurements**

We show that migration experiments that we used are robust in different assay conditions and the conclusions of our study are robust to assay conditions. We would like to make the following points:

2D migration assays have many shortcomings and like many other in vitro assays, it is far from perfect. However, we explain below why the 2D assay we used is a good in vitro system to model cancer migration: they can capture clinical and pathological parameters, and are robust with other migration assays. Finally, we also detail robustness of in silico in our findings.

We use live cell imaging-based random cell migration assays for measuring migration values in 43 breast cancer cell lines, which has often been extensively used in the research community^10–16^ and has been validated by previous work^17–22^ including ours (Van De Water’s lab). In addition, we provide two additional analyses reinforcing 2D assays can capture clinical and pathological parameters:

1. Figure S3a shows that the experimentally measured migration values in the various breast cancer cell lines using the live cell imaging-based random cell migration assays. We see that Basal (Triple negative breast cancer) cell lines are much more motile than Luminal (Wilcoxon test, P<0.00016) and Her2 positive (Wilcoxon test, P<0.0021) cell lines as expected. Since we know that breast cancer patients with Basal subtypes are highly metastatic^23^, the cell line measurements using the live cell imaging-based random cell migration assays recapitulates what we expect physiologically in the clinic.
2. We show that the migration values measured using live cell imaging-based random cell migration assays are robust in another standard 2D migration assay which is the wound healing assay. In Figure S4, we show that Hs578t cell line closes much faster the created wound than the MDA-MB-231 cell line, which is reproducible with the highest speed of Hs578t cells measured in the live cell imaging-based random cell assay. That is even though both Hs578t and MDA-MB-231 are known to be highly migratory, we see that Hs578t has more motility than MDA-MB-231 in two assay conditions. This shows that the relative ranking of the cell lines is representative of the intrinsic motility capacity of the breast cancer cell lines and hence the CellToPhenotype predictors will not be affected by the assay conditions.

Regarding the robustness of our conclusions: We found that 2D migration assays could predict patient survival, is based on two different predictors: (a) CellToPhenotype which uses live cell imaging-based random cell migration assays; and (b) siRNA-based predictor which uses Phagokinetic track (PKT) assays. Since we independently arrived at the same conclusion by using two different experimental (imaging and PKT) assays, it enhances the robustness of our conclusions.

Not only the migration assays but also *in vitro* proliferation assays suffer from disparities *in vivo* phenotype^24^. The central basis of our story is: given experimentally measured migration values in cell lines from a standard assay commonly used in the research community (irrespective of whether they are good or bad), can we learn signatures to effectively predict migration levels in patients; and are such predictions associated with patient survival? Our study finds that to be true.

**Circulating tumor cells have high migration levels**

We applied the CellToPhenotype predictors to predict the migration and proliferation levels in 5 samples of circulating breast tumor cells (CTCs) in GSE45965 data^25^, and compared it with the 110 normal breast samples and 1043 cancerous samples from breast cancer TCGA data. While predicting migration and proliferation levels in GSE45965 data, we overlapped the survival associated genes in METABRIC dataset with the genes in the GSE45965 data, for building models using CellToPhenotype predictors. We find that CTC samples have significantly higher migration levels than both the TCGA cancer samples (P<3.81e-4) and the healthy adjacent samples (Wilcoxon rank sum, P<8.34e-5). The CTC samples have significantly higher proliferation levels than the non-cancerous samples (P<9.47e-4), but not significantly higher than the cancer samples (P<0.71, Figure S6).

One weakness of this analysis is that there we have only 5 CTC samples, and therefore we carried out some additional statistical tests for the sake of robustness. We repeated the analysis using an ANOVA test^26^ to find similar results. An ANOVA test between predicted migration levels for CTC samples are higher compared to both cancers (P<5.9e-08) and healthy adjacent samples (P<2.2e-16). An ANOVA test between predicted proliferation levels for CTC samples are higher compared to non-cancerous samples (P<2.2e-16) but not with cancer samples (P<0.72). These findings are similar to what we obtained using a Wilcoxon test in Figure S6. A two-sample Fisher-Pitman permutation test^27,28^ showed a significant difference between the means of predicted migration of CTC samples with cancer samples (P<7.13e-08), and between CTC samples with normal samples (P<2.2e-16). There is also a significant difference between the means of the predicted proliferation of CTC samples with normal samples (P<2.2e-16), and there is no significant difference between CTC samples with cancer samples (P<0.72).

**References**

1. Barretina, J. *et al.* The Cancer Cell Line Encyclopedia enables predictive modelling of anticancer drug sensitivity. *Nature* **483**, 603–607 (2012).

2. Therneau, T. A Package for Survival Analysis in S. R package version. *Survival* (2012).

3. Friedman, J., Hastie, T. & Tibshirani, R. Regularization Paths for Generalized Linear Models via Coordinate Descent. *J. Stat. Softw.* **33**, (2010).

4. Freedman, D. A. *Statistical models: Theory and practice*. *Statistical Models: Theory and Practice* (2009). doi:10.1017/CBO9780511815867

5. Tibshirani, R. Regression Shrinkage and Selection via the Lasso Robert Tibshirani. *J. R. Stat. Soc. Ser. B* (1996). doi:10.1111/j.1467-9868.2011.00771.x

6. Homrighausen, D. & McDonald, D. The lasso, persistence, and cross-validation. in *Proceedings of the 30th International Conference on Machine Learning* (2013).

7. Wang, S., Nan, B., Rosset, S. & Zhu, J. Random lasso. *Ann. Appl. Stat.* (2011). doi:10.1214/10-AOAS377

8. Chattefuee, S. & Hadi, A. S. *Regression Analysis by Example*. *John Wiley & Sons.* (2015). doi:10.1002/0470055464

9. Bilal, E. *et al.* Improving Breast Cancer Survival Analysis through Competition-Based Multidimensional Modeling. *PLoS Comput. Biol.* **9**, (2013).

10. Van Roosmalen, W. *et al.* Tumor cell migration screen identifies SRPK1 as breast cancer metastasis determinant. *J. Clin. Invest.* **125**, 1648–1664 (2015).

11. Mathieu, E. *et al.* Time-lapse lens-free imaging of cell migration in diverse physical microenvironments. *Lab Chip* (2016). doi:10.1039/c6lc00860g

12. Rajesh Kumar, M. & Joice Sophia, P. Nanoparticles as precious stones in the crown of modern molecular biology. in *Trends in Insect Molecular Biology and Biotechnology* (2018). doi:10.1007/978-3-319-61343-7_16

13. Peeters, M. C. *et al.* The adhesion G protein-coupled receptor G2 (ADGRG2/GPR64) constitutively activates SRE and NFκB and is involved in cell adhesion and migration. *Cell. Signal.* (2015). doi:10.1016/j.cellsig.2015.08.015

14. Van Roosmalen, W., Le Dévédec, S. E., Zovko, S., De Bont, H. & Van De Water, B. Functional screening with a live cell imaging-based random cell migration assay. *Methods Mol. Biol.* **769**, 435–448 (2011).

15. Tasdemir, N. *et al.* Comprehensive phenotypic characterization of human invasive lobular carcinoma cell lines in 2D and 3D cultures. *Cancer Res.* (2018). doi:10.1158/0008-5472.CAN-18-1416

16. Meyer, A. S. *et al.* 2D protrusion but not motility predicts growth factor-induced cancer cell migration in 3D collagen. *J. Cell Biol.* (2012). doi:10.1083/jcb.201201003

17. Naffar-Abu-Amara, S. *et al.* Identification of novel pro-migratory, cancer-associated genes using quantitative, microscopy-based screening. *PLoS One* (2008). doi:10.1371/journal.pone.0001457

18. Herber, R. L. & Hulkower, K. I. Cell Migration and Invasion Assays as Tools for Drug Discovery. *Pharmaceutics* (2011). doi:10.3390/pharmaceutics3010107

19. Lavelin, I. *et al.* Discovery of novel proteasome inhibitors using a high-content cell-based screening system. *PLoS One* (2009). doi:10.1371/journal.pone.0008503

20. Le Dévédec, S. E. *et al.* Systems microscopy approaches to understand cancer cell migration and metastasis. *Cellular and Molecular Life Sciences* (2010). doi:10.1007/s00018-010-0419-2

21. Van Roosmalen, W. *et al.* Tumor cell migration screen identifies SRPK1 as breast cancer metastasis determinant. *J. Clin. Invest.* (2015). doi:10.1172/JCI74440

22. Le Dévédec, S. E., Lalai, R., Pont, C., De Bont, H. & Van De Water, B. Two-photon intravital multicolor imaging combined with inducible gene expression to distinguish metastatic behavior of breast cancer cells In Vivo. *Mol. Imaging Biol.* (2011). doi:10.1007/s11307-010-0307-z

23. Chikarmane, S. A., Tirumani, S. H., Howard, S. A., Jagannathan, J. P. & Dipiro, P. J. Metastatic patterns of breast cancer subtypes: What radiologists should know in the era of personalized cancer medicine. *Clinical Radiology* (2015). doi:10.1016/j.crad.2014.08.015

24. Gao, H. *et al.* High-throughput screening using patient-derived tumor xenografts to predict clinical trial drug response. *Nat. Med.* (2015). doi:10.1038/nm.3954

25. Lang, J. E. *et al.* Expression profiling of circulating tumor cells in metastatic breast cancer. *Breast Cancer Res. Treat.* **149**, 121–131 (2015).

26. Cuevas, A., Febrero, M. & Fraiman, R. An anova test for functional data. *Comput. Stat. Data Anal.* (2004). doi:10.1016/j.csda.2003.10.021

27. Hothorn, T., Hornik, K., Wiel, M. A. van de & Zeileis, A. Implementing a Class of Permutation Tests: The **coin** Package. *J. Stat. Softw.* (2008). doi:10.18637/jss.v028.i08

28. Neuhäuser, M. & Manly, B. F. J. The Fisher-Pitman Permutation Test When Testing for Differences in Mean and Variance. *Psychol. Rep.* (2004). doi:10.2466/pr0.94.1.189-194

**Supplementary Figures**


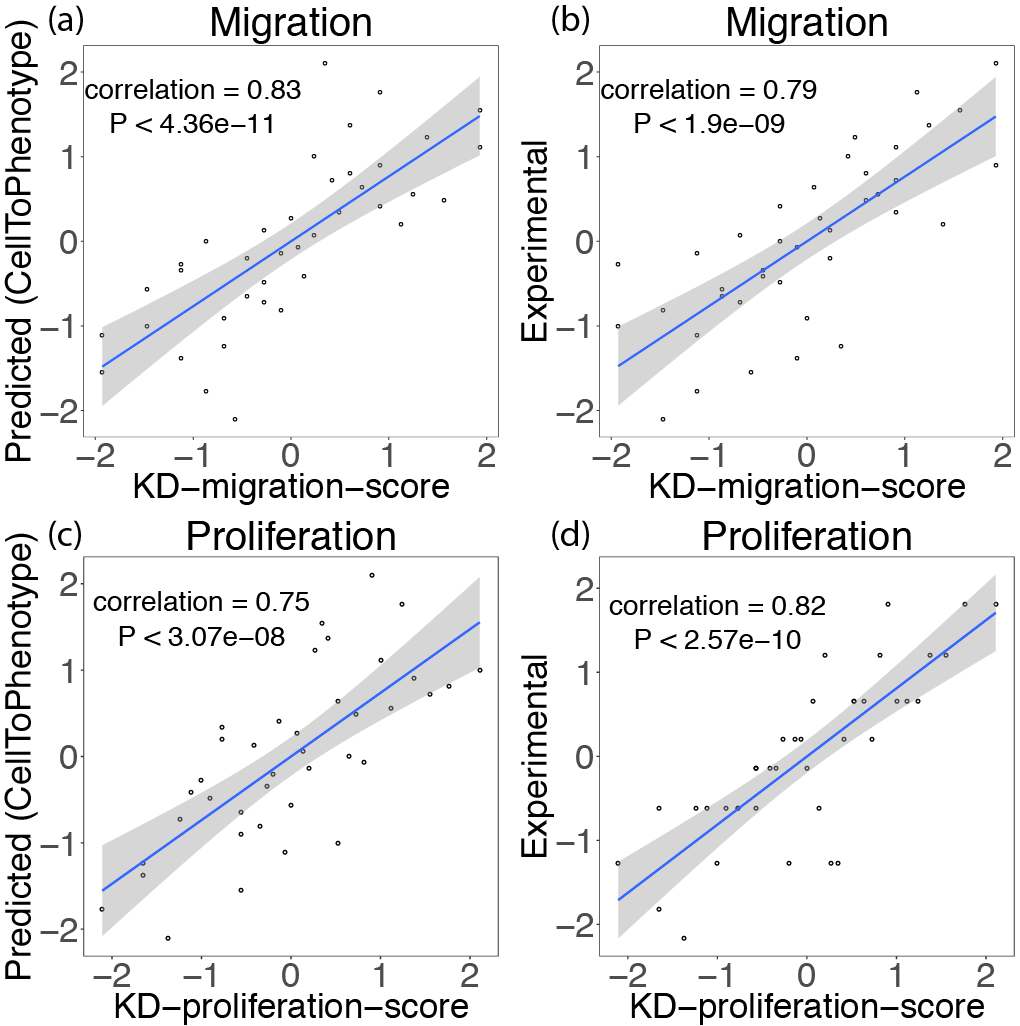


***Figure S1:*** *Spearman correlation between KD-migration-score:* ***(a)*** *with predicted migration levels (CellToPhenotype);* ***(b)*** *with experimentally measured migration values, on 40 breast cancer cell lines. Spearman correlation between KD-proliferation-score:* ***(c)*** *with predicted proliferation levels;* ***(d)*** *with experimentally measured proliferation values.*


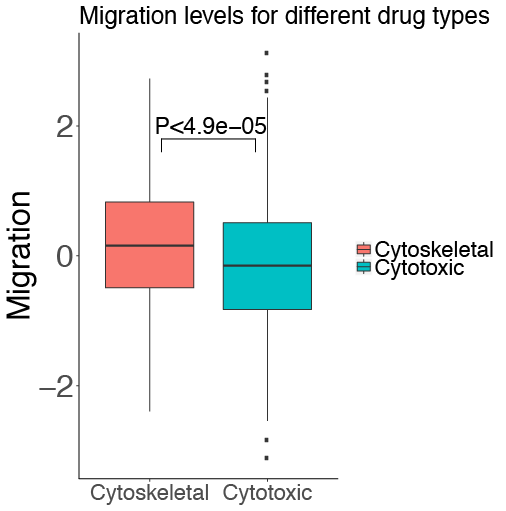


***Figure S2:*** *Box plots showing estimated migration levels are higher for the patient treated with cytoskeletal drugs for the patients treated only with cytotoxic drugs (Wilcoxon rank-sum, P<4.9e-5).*

***Figure S3****: (a) Experimentally measured migration values in various breast cancer subtypes (cell-lines). (b) Experimentally measured proliferation values in various breast cancer subtypes (cell-lines). (c) Predicted migration levels in various breast cancer subtypes (cell-lines). (d) Predicted proliferation levels in various breast cancer subtypes (cell-lines). We see that the predicted and experimentally measured migration values behave similarly across various breast cancer subtypes (similar results for proliferation).*


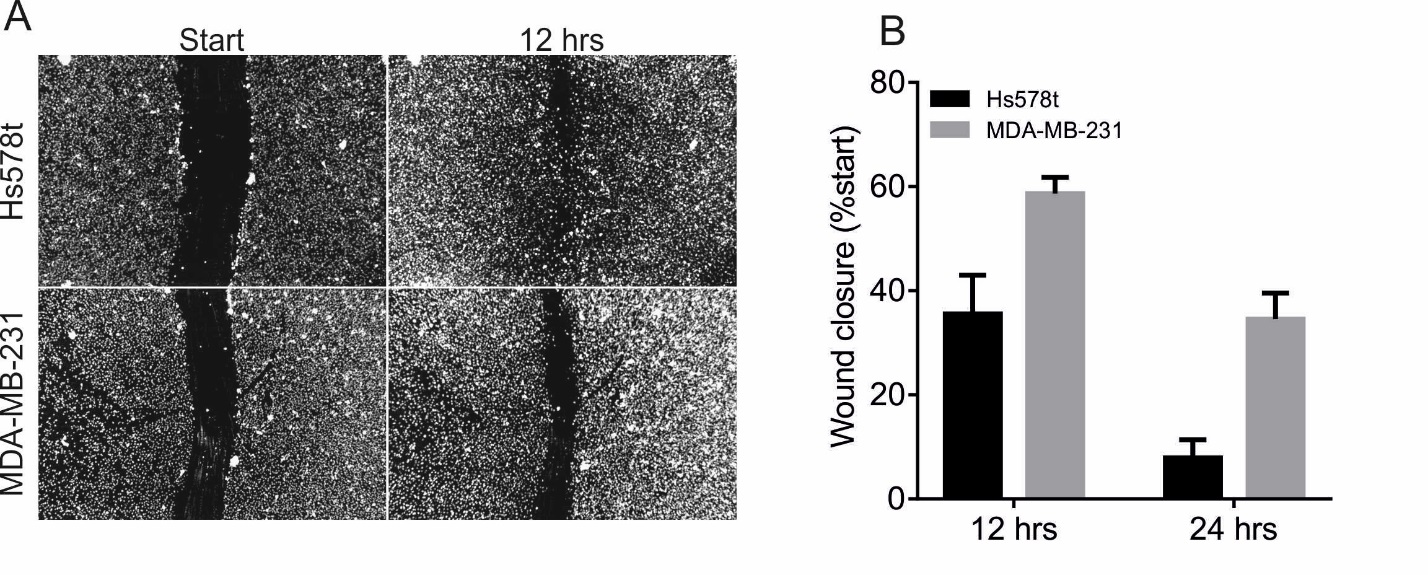


***Figure S4:*** *Wound healing assay of both basal B Hs578t and MDA-MB-231 cell lines show that Hs578t closes the wound much faster than MDA-MB-231 at similar cell density. Bar graph represent the standard error of the mean (SEM, n=12 per cell line).*

***Figure S5:*** *Predicted migration (M) and Proliferation (P) levels using CellToPhenotype (LASSO-based) predictors for a single patient for 10 iterations. The predicted values are quite consistent over various iterations.*

***Figure S6:*** *Predicted migration (M), proliferation (P) levels of 5 samples of circulating tumor cells (CTC) from GSE45965 data, compared with the 110 noncancerous samples and 1043 breast cancer TCGA samples.*
